# Effectiveness of a high-intensive trauma-focused, family-based therapy for youth exposed to family violence: study protocol for a randomized controlled trial

**DOI:** 10.1186/s13063-021-05981-4

**Published:** 2022-01-17

**Authors:** Valerie Fictorie, Caroline Jonkman, Margreet Visser, Marjolein Vandenbosch, Majone Steketee, Carlo Schuengel

**Affiliations:** 1 Vrije Universiteit Amsterdam, Amsterdam, The Netherlands; 2 Children’s Trauma Centre of Kenter Youthcare, Amsterdam, The Netherlands; 3 Verwey-Jonker Institute, Utrecht, The Netherlands

**Keywords:** Family violence, Domestic violence, Posttraumatic stress, Intensive, Children, Parent, Security, Safety, Trauma, Relationship

## Abstract

**Background:**

Family violence is a common problem with direct adverse effects on children as well as indirect effects through disruption of parenting and parent-child relationships. The complex interrelationships between family violence, parenting, and relationships make recovery from psychological responses difficult. In more than half of the families referred to mental health care after family violence, the violence continues. Also, the effect sizes of “golden standard” treatments are generally lower for complex trauma compared to other forms of trauma. In the treatment of complex trauma, trauma-focused therapies including cognitive restructuring and imaginal exposure are most effective, and intensifying therapy results in faster symptom reduction. Furthermore, there is promising evidence that adding a parental component to individual trauma treatment increases treatment success. In family-based intensive trauma treatment (FITT), these factors are addressed on an individual and family level in a short period of time to establish long-term effects on the reduction of trauma symptoms and recovery of security in the family. This randomized controlled multicentre study tests if FITT is an effective treatment for concurrent reduction of trauma symptoms of children, improvement of parenting functioning, and increasing emotional and physical security in children, through the improvement of parent-child relationships.

**Methods:**

The effectiveness of FITT will be tested by a RCT design. A total of 120 adolescents with a history of family violence and PTS symptoms will be randomized to (a) an intensive trauma treatment with a parent and systemic component (FITT), (b) an intensive trauma treatment without these components (ITT), and (c) treatment as usual (TAU, low-frequency trauma treatment with parent therapy and family sessions). Changes in children’s trauma symptoms, child and parent functioning, and emotional and physical security in the family will be monitored before, during, after, and at 3 months follow-up.

**Discussion:**

Comparing these interventions with and without a high intensive frequency and parenting and family components can help to understand if and how these interventions work and can contribute to the ambition to recover from the impact of family violence and restore emotional and physical security for children and young people.

**Trial registration:**

Netherlands Trial Register Trial NL8592. Registered on 4 May 2020

## Administrative information

Note: the numbers in curly brackets in this protocol refer to SPIRIT checklist item numbers. The order of the items has been modified to group similar items (see http://www.equator-network.org/reporting-guidelines/spirit-2013-statement-defining-standard-protocol-items-for-clinical-trials/).

**Table:** 

Title {1}	Effectiveness of a high intensive trauma-focused, family-based therapy for youth exposed to family violence: Study protocol for a randomized controlled trial
Trial registration {2a and 2b}.	Netherlands Trial Register Trial NL8592, Registered 4 May 2020. https://www.trialregister.nl/trial/8592
Protocol version {3}	8 July 2021, version 1.0
Funding {4}	The Netherlands Organisation for Health Research and Development (ZonMW) funds this study through a research grant (70-74900-98-002). Kenter Jeugdhulp provides additional financial support.
Author details {5a}	V.Fictorie, VU Amsterdam and Children’s Trauma Centre of Kenter Youthcare and VU University C.S. Jonkman, VU Amsterdam and Kenter Youthcare M.M. Visser, Children’s Trauma Centre of Kenter Youthcare M.M.L.J.Z.Vandenbosch, VU Amsterdam M. Steketee, Verwey-Jonker Institute C. Schuengel, VU Amsterdam
Name and contact information for the trial sponsor {5b}	VU Amsterdam **VU FGB – Child and Family Studies** Van der Boechorststraat 7 1081 BT Amsterdam
Role of sponsor {5c}	VU Amsterdam is the sponsor and responsible for the study design, data collection, data management, data analysis and interpretation of the data, and writing and submitting reports for publication. The funder (ZonMW) monitors the project through yearly reports and evaluations. The funder has no role in the study design and collection, analysis, and interpretation of the data.

## Introduction

### Background and rationale {6a}

#### The prevalence and impact of family violence

Family violence is a common problem and affects children and their families. In the Netherlands, almost 6% of the population aged 18 and older experienced some incident of family violence over the 5 years prior to the study [1]. In 2018, it was estimated that between 26 and 37 children and youth up to age 18 years per 1000 were victims of child maltreatment; in almost half of these cases, child maltreatment happened in a context of family violence, mostly between parents [2]. Family violence has a strong adverse impact on children, both immediately and in the long term, across somatic and psychological domains [3]. Children can experience problems in multiple domains of functioning [4] and are at risk to develop posttraumatic stress (PTS) symptoms [5]. Besides direct effects of exposure to family violence, the association of family violence with parenting and the parent-child relationship indirectly increases the risk of maladaptive developmental pathways (5–7). Family violence has an impact on parents’ mental health, such as PTS disorder [6] and depression [7]. Parental PTS can mediate the effect of violence on parenting behaviour [8], and higher levels of parental stress are associated with higher levels of children’s PTS [5]. The influence of family violence on children, parents, and thereby on parenting and the parent-child relationship makes family violence a complex problem. Effect sizes of “golden standard” trauma treatments are generally lower for childhood trauma like family violence than for other forms of trauma [9]. With regard to long-term safety in the family, in more than half of the families referred to mental health care after family violence, the violence was still severe or occurred frequently one and a half years later [10]. This indicates a need for interventions for the whole family system that address long-term safety.

#### Direct pathway: the effects of family violence on children

Family violence has a direct effect on children’s functioning. After exposure to family violence children can develop posttraumatic stress symptoms, anxiety, and depressive symptoms [11, 12]. Almost a quarter of young children exposed to family violence develop posttraumatic stress (PTS) symptoms at clinical levels and another 16% in the subclinical range [5]. In a referred sample, 21% of children exposed to family violence had clinical posttraumatic stress symptoms [13]. Children’s direct responses to violence have been conceptualized using cognitive context theory [14]. According to this theory, violence is perceived as a stressor, leading to cognitive appraisal and selection and generation of coping responses. Contextual, cognitive, and developmental factors determine children’s appraisal and ensuing responses to family violence. For example, children who consider the violence as a negative event and attribute the conflict to themselves can experience more stress than children who consider the conflict as unimportant or attribute the conflict to others or the circumstances [14]. According to emotional security theory (EST) [15], children try to maintain a sense of safety and security within the family system, which may lead to emotional, behavioural, and cognitive short-term adaptations that may be maladaptive in the long run and outside the context of the family. In reaction to the violence, children show intense distress (emotional reactivity) [16], avoidance of or involvement in the conflict (behavioural regulation) [17, 18], and update their internal representations of the integrity of the family and the risk that they are running to become a target of violence themselves (cognitive response) [19]. Children can become more vulnerable to psychological problems if they experience prolonged difficulties in achieving and maintaining safety and security within the family system [20].

#### Indirect pathway: the effect of family violence on parents and family relationships

In addition to direct effects, family violence may also indirectly cause emotional and behaviour problems in children through parenting and family relationships. According to the EST, family violence leads to parenting difficulties that negatively influence the parent-child relationship and the representation of the parent-child relationship and undermine children’s well-being. Exposure to family violence has been linked to more negative and less positive parenting compared to parenting in non-violent families [21]. Parents involved in family violence showed more harsh discipline towards their children [21], were less supportive, showed less effective parenting [22], were less emotionally available [23, 24], and used more psychological control towards their children [25, 26]. These parenting difficulties make it difficult for children to see their parents as a source of protection and support. For example, maladaptive parenting mediates the relationship between family violence and children’s internalizing problems [27]. Furthermore, there is evidence for the association of family violence with a disturbed parent-child interaction [23, 24]. High levels of parental unavailability in combination with high levels of family violence are associated with insecure representations of the parent-child relationship, and these representations are associated with children’s engagement difficulties at school [24]. Also, the magnitude of the association between family violence and children’s internalizing problems is dependent on the degree of parental warmth and availability [27].

#### Treatment after family violence

International guidelines for children (AACAP, 2010; NICE, 2018) recommend individual trauma-focused cognitive behavioural therapy (TF-CBT) and eye movement desensitization and reprocessing (EMDR) after traumatic events, like family violence. On average, TF-CBT and EMDR are effective in addressing PTS symptoms of children [28]. However, treatment effect sizes were generally lower for different forms of childhood maltreatment like family violence, compared to single-event trauma [9]. Furthermore, most studies on complex trauma focused on TF-CBT for adults instead of children, and in general, the number of trials with EMDR was limited. In contrast to EMDR, TF-CBT is a multicomponent treatment consisting of non-trauma-focused and trauma-focused components. When treating adults with complex trauma, trauma-focused therapies that include cognitive restructuring and imaginal exposure are most effective [29]. In line with this research, a Dutch study [30] recently showed that (a) exposure therapy is effective in the treatment of adults with complex trauma and (b) adding a non-trauma-focused component (affective and interpersonal regulation training) to the exposure therapy did not result in better outcomes. Interestingly, intensifying the exposure therapy resulted in faster symptom reduction. The faster reduction of symptoms is especially important for youth because posttraumatic stress hinders development [4] and adolescence is a critical period for social development, including peer influence [31]. There are also promising results in intensive trauma-focused treatment for adults that combined EMDR and imaginal exposure [32] and for adolescents who received intensive exposure therapy [33]. The characteristic of these intensive treatment programs is that the trauma treatment is given by different therapists. Therapist rotation is thought to decrease fear and avoidance behaviour of the therapist [34] and thereby increase treatment fidelity.

Besides offering trauma-focused therapy and intensifying the frequency in which these therapies are offered, trauma treatment might be enhanced by including trauma-focused bodily oriented therapy [35]. After trauma, children can experience flashbacks and intense emotions like fear, bodily sensations, and physical complaints [36]. According to Lang’s informational theory [37, 38] fear is an associated network of information based on previous experiences, developed to avoid or flight from danger in order to increase survival. This network is activated when confronted with a stimulus that matches information stored in this network. Based on these previous experiences, this stimulus is associated with a specific meaning and physiological and behavioural response [39]. For example, for someone who experienced a life-threatening fire, the sound of a siren could be a stimulus associated with danger and response elements like racing heart, muscle tensing, and hiding. These responses once were adaptive and promoted survival during the traumatic events but are now maladaptive [36]. Bodily oriented therapy, developed from clinical practice, is focused on the affective and behavioural components of these habitual responses. Combining trauma therapy with physical activities has been found associated with lower PTSD symptoms compared to trauma therapy without physical activities [40]. One randomized trial found that trauma-informed yoga reduced PTS symptoms [41]. These findings justify the inclusion of bodily oriented intervention components alongside other trauma-focused components.

Trauma-focused therapeutic components like EMDR and exposure may reduce PTS but may not automatically lead to improvements in parent functioning and the parent-child relationship. To achieve this, parental and systemic components are added to treatment programs like TF-CBT. Especially in situations of family violence that affect the whole family system, it is important to examine the value of adding parental and systemic components to EMDR and exposure. Meta-analytic data shows that interventions to reduce child maltreatment yielded larger effect sizes if focused on parental skills and social/emotional support than interventions without such focus [42]. Evidence that adding a parental component to individual trauma treatment increases treatment success, measured in children’s PTS symptoms, is also promising, yet inconclusive [28]. Few studies have systematically reviewed and/or meta-analytically examined the effectiveness trials that included interventions focusing on parenting factors in situations of family violence.

#### The added value of parent training and parent-child sessions

A systematic literature search (Fictorie V, Jonkman CS, Visser, MM, Steketee, M & Schuengel, C: An umbrella review on the added value of parent training and parent-child sessions in the treatment of family violence: how to promote improvement of parenting behaviour, the parent-child relationship and structural family safety, in preparation) revealed a limited but growing body of research on how parenting training and parent-child sessions may lead to changes in parenting behaviour, in the parent-child relationship, and especially in family safety. Four systematic reviews were identified on parenting-focused interventions for violence in families [43–46]. One meta-analysis [45] concluded that parent-child or parent intervention positively affects positive parenting behaviour and children’s functioning. There was no difference in effect between parent versus combined parent and child intervention but statistical power was too low. Another review [46] concluded that children with outside support fared better on behaviour problems, that improvement was stronger when offering more than five contacts to the family, and that changes in parenting behaviour and psychiatric symptoms were largely responsible for treatment effects on children’s conduct problems. It was not possible to draw unambiguous conclusions about how parent- and family-focused interventions affect parent functioning, child functioning, and the parent-child relationship due to the limitations of the reviewed studies. These limitations included the relatively large variation across studies in the manner that interventions were offered, in the types of intervention offered, and in the target of the intervention and outcome measures. Also, understanding the role of parenting and parent-child relationships within therapeutic interventions is hampered by methodological challenges, such as informant bias in the case of self-report instruments of parenting and relationships. Furthermore, there is a need for larger sample sizes, controlled study designs, protocolled treatments, and assessment of treatment fidelity [43]. Because of the great variations in type of interventions in the different studies, it is not possible to draw conclusions about the parental factors most important to cause changes in parent and child functioning and the parent-child relationship. There is some evidence that trauma-informed parenting interventions improving positive parenting skills are most important [45] and focusing on the support system and parental health is promising [46]. Most multicomponent treatments are focused on these factors [46]. To understand the added value of attention to parental factors in the treatment of family violence, studies are needed of (a) protocolled treatments; (b) treatments focused on specific parent components, like trauma-informed parenting, support, and parental health; (c) different types of instruments (observation, questionnaires, interview) and for multiple informants; (d) outcome measures for parenting behaviour, parent-child relationship, and family safety; (e) controlled study designs; (f) large study samples; and (g) assessment of treatment fidelity.

#### Family-based intensive trauma treatment

Because of the severe impact of family violence on children and their families and the complexity of family violence through the reciprocal influence of children’s PTS and parent and family functioning, we developed an intervention protocol that addresses these factors on an individual and family level in a short period of time: the family-based intensive trauma treatment (FITT). The goal of FITT is to establish long-term effects on the reduction of children’s trauma symptoms and recovery of security in the family. By first concurrently reducing children’s trauma symptoms and improving parenting functioning, the stage is set to increase the emotional and physical security in children through the improvement of the parent-child relationship.

FITT therefore consists of (a) intensive trauma treatment (exposure, EMDR, bodily oriented therapy) for the child; (b) individual parent therapy, based on the horizon method [47] (the Horizon-method is originally a group-based version of TF-CBT) [48], focusing on lowering parental stress and increasing trauma-sensitive and supportive parenting; and (c) family therapy focusing on sharing thoughts and feelings about the traumatic events, recognizing and breaking destructive patterns, and finding solutions for recurring problems.

#### Effectiveness study of FITT

This study examines to which extent FITT is an effective treatment for concurrent reduction of children’s trauma symptoms, improvement of parenting functioning, and enhanced emotional and physical security in children, through the improvement of parent-child relationships—in order to establish long-term effects on the reduction of trauma symptoms and recovery of security in the family. In this multi-centre study with a randomized controlled design, we compare FITT with an intensive trauma treatment without a parent and systemic component (ITT) and with treatment as usual (TAU) and low-frequency trauma treatment with parent therapy and family sessions). Comparing these interventions with and without specific components contributes to understanding if these interventions are effective and how these interventions work [49]. These insights may be used to increase treatment effectiveness [50] for adolescence with complex traumatic experiences. To further detect the mechanism of change, we included measurements during treatment [50].

### Objectives {7}

The primary objective is to examine the effectiveness of parental and systemic components that complement an intensive trauma treatment for children who have been exposed to family violence in the family. This objective translates into the following research question:

1. Does intensive trauma therapy for children with a family-based component (FITT) lead to a stronger reduction of trauma symptoms of children and a stronger increase of structural emotional safety than intensive trauma therapy without a family-based component (ITT)?

In addition, secondary objectives regard the elucidation of the intervention mechanism and effects on secondary outcomes. These objectives lead to the following research questions:

1. Does intensive trauma therapy for children with a family-based component (FITT) lead to faster reduction of trauma symptoms and increase of structural emotional safety than low-frequency trauma treatment with parent therapy and family sessions (TAU)?
  - After 4 weeks of treatment, does intensive trauma therapy for children with a family-based component (FITT) lead to a stronger reduction of trauma symptoms and a stronger increase of structural emotional safety than low-frequency trauma treatment with parent therapy and family sessions (TAU)?
  - After the end of treatment, does intensive trauma therapy for children with a family-based component (FITT) lead to an equal reduction of trauma symptoms and an equal increase of structural emotional safety as in low-frequency trauma treatment with parent therapy and family sessions (TAU)?
2. At the end of treatment, to what extent do we find the differences between intensive trauma therapy for children with a family-based component (FITT) and intensive trauma therapy without this family-based component (ITT) in trauma-informed parenting?

### Trial design {8}

This multicentre study is a parallel-group, randomized controlled component trial to examine (1) the addition of a component with parenting therapy and family sessions to an intensive trauma-focused therapy component and (2) the addition of an intensive component to a low-frequent trauma-focused therapy with parenting therapy and family sessions. This study is a superiority trial comparing FITT and TAU after 4 weeks of treatment and FITT and ITT at end of treatment and follow-up. The comparison between FITT and TAU at follow-up concerns an equivalence trial. This randomized controlled design includes follow-up with 96 (completer’s rate 80%) children and their caregiver(s) divided over two intensive trauma treatment arms and one treatment as a usual arm. The allocation rate for all comparisons is 1:1. The effectiveness trial comprises a pre-treatment, during treatment, end of treatment, and follow-up design.

## Methods: participants, interventions, and outcomes

### Study setting {9}

This multicentre research study takes place in four different clinical health care settings for youth in the Netherlands: Child and Youth Trauma Centre Kenter Jeugdhulp, GGZ Rivierduinen, UMC Utrecht, and Arq.

### Eligibility criteria {10}

Children are eligible to participate in the study if they meet the following inclusion criteria: (a) the child has been exposed to family violence; (b) the acute safety in the family has been established; (c) the child is between 12 and 20 years old; (d) the child lives at home with its caregiver(s); (e) caregivers, living with the child can participate in the systemic components; (f) the child has posttraumatic stress symptoms, at least intrusions and avoidance; and (g) caregivers and children master the Dutch language. The exclusion criteria for participating in the study are (a) children and caregivers who have acute psychotic symptoms or severe alcohol and drug addictions and (b) children with a suicide attempt in the past 3 months. The in- and exclusion criteria are established with an intake interview with a trained therapist and standardized questionnaires.

All therapists participating in the study are trained in the overall treatment program and have additional training in the part of the treatment program they will perform. Exposure therapy will be performed by therapists who have basic training in cognitive behavioural therapy. EMDR therapy will be performed by the therapists who completed the advanced EMDR training (VEN, Dutch EMDR Association). The bodily oriented therapy is offered by psychomotor therapists or therapists with experience in trauma and bodily oriented therapy under the supervision of an experienced psychomotor therapist. Parent therapy is performed by a family therapist or family worker. Together with a child therapist, the family therapist/worker will also offer the family sessions.

### Who will take informed consent? {26a}

Children between the age of 12 and 20 years old and referred to one of the participating trauma centres after being exposed to family violence will be given a brochure about the study, so are their parents. Subsequently, the therapist asks the family permission to share their contact details with the researcher. After approval, the therapist will send the researcher the contact details by email. Within two working days, the researcher will contact the family members separately to explain the study. An information letter with detailed information about the study will be sent to the families before the first intake interview at the respective centre. The families will have at least 1 week to consider their decision about participating in the study. If the family agrees to participate, informed consent will be sought from both parents, and children during the first intake. After the inclusion criteria are met and informed consent is obtained, participating families will be randomly assigned to one of the treatment conditions by an independent researcher using a computerized randomization procedure with lottery drawings.

### Additional consent provisions for collection and use of participant data and biological specimens {26b}

Participants will be informed that if they withdraw from the trial, their previously collected data will be used for the study. In the consent form, participants will be asked permission for the insight into their data, when relevant, by other people from the research team and regulatory authorities that are mentioned in the information letter. Participants will also be asked if the research team may contact them for future studies. The trial does not involve the collection of biological specimens.

### Interventions

#### Explanation for the choice of comparators {6b}

Intensive trauma therapy (ITT) is the comparator of family-based intensive trauma treatment (FITT) to examine the addition of a component with parenting therapy and family sessions to an intensive trauma-focused therapy component. It is important to understand if this addition is more effective in reducing trauma symptoms, improving parenting functioning, and increasing emotional and physical security in children, through the improvement of parent-child relationships—and thereby establishing long-term effects on the reduction of trauma symptoms and continuation of security in the family.

The second comparator for FITT is treatment as usual (TAU) to examine the addition of an intensive component to a low-frequent trauma-focused therapy with parenting therapy and family sessions. This comparator examines if FITT is as effective as TAU at the end of treatment and if FITT results in faster symptom reduction. In the view of adolescence as a critical period for development and peer influence [51], this faster symptom reduction is of great importance.

#### Intervention description {11a}

##### Components of ITT

ITT is a 3-week intensive trauma treatment program and contains a preparation phase in week 1 and an intensive phase in weeks 2 and 3.

#### Preparation phase: psycho-education and clustering

The preparation phase consists of two treatment days. On the first treatment day, the adolescent receives a 90-min online and interactive session of psycho-education about the impact of trauma and the treatment program. The aim is to inform the adolescent about (the consequences of) family violence, trauma and PTS disorder, stress regulation, the window of tolerance, and the treatment program. On the second treatment day, the adolescent is offered a 90-min session in which the different traumatic events are clustered. The adolescent and the therapist choose the six most traumatic memories, ordered by the degree to which they cause flashbacks, nightmares, and avoidance behaviour and elicit the most tension. These traumatic events will be the focus of the exposure and EMDR sessions.

#### Intensive phase: exposure, EMDR, and bodily oriented therapy

In the intensive phase, the adolescent has three treatment days per week. Each treatment day has a fixed program consisting of the following:

- 90-min Exposure
- 60-min bodily oriented therapy
- 90-min EMDR

##### Exposure

The exposure and EMDR in the program will be given by different therapists. One traumatic memory each day will be treated with exposure and EMDR. The exposure therapy is based on the Dutch translation [52, 53] of Foa’s prolonged exposure protocol [54]. Prolonged exposure therapy is a form of cognitive behavioural therapy in which the adolescent repeatedly describes the traumatic event in detail and present tense, focusing on the anxiety-provoking parts of the traumatic event. Every 5 to 10 min, the therapist checks the level of disturbance experienced by the adolescent. The adolescent learns fearful expectations stay away when being exposed to the trauma, and there is no need for avoidance behaviour.

##### Bodily oriented therapy

Between the sessions, exposure and EMDR individual bodily oriented therapy are offered by psychomotor therapists or therapists with experience in trauma and bodily oriented therapy under the supervision of an experienced psychomotor therapist. Through physical activities, the therapist supports the adolescent to:

- Enhance the understanding of the association between the body and brain
- Enhance the understanding of the association between trauma and the body
- Improve stress-regulation skills
- Recognize the effects of trauma on the body
- Calm the brain and body to prepare for the EMDR session

The therapist will work with bodily oriented exercises to achieve these goals.

##### EMDR

For the EMDR session, the most recent Dutch EMDR protocol for children and youth will be used, and all therapists completed the basic and advanced EMDR training of the Dutch EMDR Association. During EMDR therapy, the adolescent is asked to focus on the most disturbing image of the traumatic memory, and the therapist asks questions about the thoughts and feelings associated with the traumatic image and the level of disturbance. The therapist will ask the adolescent to focus on the image and the associated thoughts and feelings and at the same time focus on a distracting stimulus. This will bring a stream of associations in the form of thoughts, feelings, images, and bodily sensations and helps to reprocess the traumatic memory. The therapist will then ask what the adolescent notices and repeat the distracting stimulus. After a while, the adolescent will notice the level of disturbance will lower until the traumatic memory is desensitized. If necessary, the therapist can add cognitive interweaves during the desensitization phase to elicit new associations. An overview of ITT is presented in Table 1.

**Table 1:** Overview of the intensive trauma treatment

Intensive trauma treatment (ITT)		
	Intervention	Adolescent
Week 1: preparation phase	Psycho-education	X
	Clustering	X
Week 2: intensive phase	Exposure	X
	Bodily oriented therapy	X
	EMDR	X

#### Additions in the preparation phase

##### Psycho-education for family and network

In FITT, the session psycho-education is together with the parents and the support system. This is a 90-min online and interactive session of psycho-education about the impact of trauma and the treatment program. The aim is to inform the adolescent and family system about (the consequences of) family violence, trauma and PTS disorder, stress regulation, the window of tolerance, trauma-sensitive parenting, and resilience and the treatment program.

#### Additions in the intensive phase

##### Parent therapy

In the intensive phase in weeks 2 and 3, parents receive three 60-min sessions of individual parent therapy per week from one family therapist or family worker. In dialogue, different topics are discussed: parental stress, support system, trauma-sensitive parenting, and traumas within the family. The parent therapy and EMDR take place simultaneously, and after these sessions, there is a short parent-child interaction moment where the adolescents share their thoughts and feelings on which the parents can practice supportive and trauma-sensitive parenting. The aim of the parent therapy is to:

- Lower levels of parental stress
- Increase parental support
- Increase knowledge about trauma-sensitive parenting and increase trauma-sensitive parenting skills
- Increase the insight that the family has a joint family story in which the different family members have their own memories and meaning about the traumatic events.

#### Additional integration phase

#### Family therapy

The integration phase consists of three 90-min sessions of family therapy. Two therapists support the family to talk about the experienced family violence and current conflicts. The goal of family therapy is to:

- Create a shared trauma narrative
- Recognize destructive communicative patterns
- Recognize emotional individual vulnerabilities and survival behaviour and their mutual effects in family relationships
- Improve skills to prevent escalations and cope with destructive patterns

In Table 2, an overview of FITT is presented.

**Table 2:** Overview of the family-based intensive trauma treatment

Family-based intensive trauma treatment (FITT)					
	Intervention	Adolescent	Parents	Family	Network
Week 1: preparation phase	Psycho-education	X	X	X	X
	Clustering	X			
Weeks 2 and 3: intensive phase	Exposure	X			
	Bodily oriented therapy	X			
	EMDR	X			
	Parent therapy		X		
Week 4: integration phase	Family therapy	X	X	X	

##### Components of TAU

TAU is comparable across the four trauma centres and consists of low-frequent trauma-focused therapy with parenting therapy and family sessions. In contrast to the high intensity of FITT, TAU is offered about once a week and without therapist rotation. In TAU, the adolescents receive therapy from one therapist and parents receive therapy from another therapist. This individual or group-based trauma-focused cognitive behavioural therapy for adolescents and their parents is focused on the following components:

- Psycho-education trauma and PTSD: increasing knowledge about (the consequences of) trauma and family violence and trauma-sensitive parenting and understanding the window of tolerance
- Affect regulation and coping: learning children to recognize and regulate feelings of stress and enhancing coping skills
- Trauma processing: EMDR, exposure, writing therapy, or bodily oriented therapy
- Parenting skills and competence: enhancing trauma-sensitive parenting skills, lowering levels of parental stress, and increasing social support.
- On indication: family relationships—sharing the trauma narrative and accompanying feelings and cognitions (for example feelings of shame or guilt) and strengthening of skills to prevent escalations

#### Criteria for discontinuing or modifying allocated interventions {11b}

In accordance with the Medical Research Involving Human Subjects Act, the sponsor will suspend the study if there is plausible ground that the continuation of the study will jeopardise the subject’s health or safety. The sponsor will then notify the accredited Medical Ethical Committee (from Amsterdam UMC) without undue delay of a temporary halt including the reason for such an action. The study will be suspended pending a further positive decision by the accredited Medical Ethical Committee. The investigator will take care that all subjects are kept informed.

#### Strategies to improve adherence to interventions {11c}

To increase treatment fidelity, a training protocol for (F)ITT is developed, and the therapists receive training in the (F)ITT protocol before data collection. Furthermore, therapists receive supervision on exposure, EMDR, parent and family therapy, and bodily oriented therapy during data collection. Exposure supervision is given by a healthcare psychologist who is a licensed supervisor from the Dutch cognitive-behavioural therapy association. EMDR supervision is given by healthcare and clinical psychologist who both are licensed supervisors from the Dutch EMDR association. The supervisor for the parent and family sessions is a clinical psychologist with advanced experience in the field of trauma and family therapy. The supervisor for the bodily oriented therapy is an experienced psychomotor therapist who works with traumatized children. To monitor adherence to the treatment protocol, therapists are asked to fill in a treatment evaluation form after each therapy session.

The trial will be monitored by the independent physician of the four participating youth mental health centres. Two times per year, the independent physician will randomly check the research file of a study participant on the following:

- The involvement of a therapist registered in the Dutch register for healthcare professionals
- The presence of a safety plan in cases of suicidal thoughts/ideations and/or self-damaging behaviour
- The compliance of in- and exclusion criteria (in the first year, the first three study participants will be checked, and thereafter, every half year, a participant will be randomly checked on in and exclusion criteria)
- Serious adverse events (SAEs) and if these are reported to the independent physician, inspection for healthcare and youth, the research institute and the Medical Ethical Committee
- The presence of the cause conceptualisation

If any errors are observed, the files of all study participants within the specific centre will be checked.

To monitor the collection, storage, and processing of the data, each year, peer auditors trained in data management will randomly check the research file on the following:

- Presence of informed consents
- Inclusion pace and drop out percentage
- Presence and completeness of the research data

If any errors are observed, the files of all study participants within the specific centre will be checked.

Furthermore, daily monitoring of the study will be done by the coordinating investigator, project leader, and principal investigator. Frequent contacts with the four trauma centres and research team meetings will be held to evaluate the progress of the study. All participating trauma centres have a contact person, who will be involved in all phases of the research.

#### Relevant concomitant care permitted or prohibited during the trial {11d}

Other psychological care for the adolescent will be paused from the first measurement until follow-up. Ongoing practical support can be continued. In cases of new warning signals of unsafety or suicidal thoughts, crisis interventions are offered by the involved centre participating in this study

#### Provisions for post-trial care {30}

After the follow-up measurement, an evaluation with the family will take place according to the procedure of the participating centres. If needed, post-trial care will be offered.

### Outcomes {12}

Table 3 presents an overview of the time points for each outcome measurement.

**Table 3:** Measures

	*Measures*				Measurement			
	Measure	Items	Respondent*	Type	T(0)	T(1–11)	T(12/14)	T(13)
Child PTSD	CAPS-CA		c	Interview	*			*
	CRIES	13	cp	Questionnaire	*	*	*	*
	TSCC	54	c	Questionnaire	*		*	*
Child functioning	SDQ	25	cp	Questionnaire	*		*	*
	CDI	28	c	Questionnaire	*		*	*
	CDC	20	p	Questionnaire	*		*	*
Parent functioning	NOSI-K	25	p	Questionnaire	*	*	*	*
	Trauma-informed	6	p	Questionnaire	*		*	*
	IES-r	22	p	Questionnaire	*		*	*
	YAS-R	29	p	Questionnaire	*		*	*
	DPAS	9	p	Questionnaire	*		*	*
Structural emotional and physical safety	CTS	77	p	Questionnaire	*		*	*
	CTS-pc	27+39	c	Questionnaire	*		*	*
	PRTI	21	p	Questionnaire	*		*	*
	ACE-Q	10	p	Questionnaire	*		*	*
	New IPV incidents	8	cp	Questionnaire	*	*	*	*
	SIS	43	c	Questionnaire	*		*	*
	SIFS	7	c	Questionnaire	*		*	*
	SIM-PR	28	p	Questionnaire	*		*	*
	Security scales	15	cp	Questionnaire	*		*	*
	FIT	4 tasks	cp	Observation			*	
Total items child					232	21	232	232
Total items parent					335	46	335	335

Marked measures are part of routine outcome measuring

#### Primary outcomes

##### Child PTSD symptoms

The Clinician-Administered-Ptsd-Scale-Children-And-Adolescents (CAPS-CA) [55] is a standardized clinical interview, to assess PTSD conform the DSM-5 standards. A Dutch translation of the CAPS-CA [56] is administered to children aged 8–18. The core traumatic event is chosen based on the life events checklist. If the core traumatic event is a sequence of events, which is often the case in family violence, a brief term is chosen to capture the core of these events. The CAPS-CA assesses the frequency and intensity in which each PTS disorder symptoms occur. A 5-point severity rating scale is used for all symptoms, ranging from 0 (= absent) to 4 (= extreme/incapacitating). An example item is *In the past month, have you had upsetting thoughts, pictures or sounds of what happened come into your mind when you didn’t want them to?*. A total symptom severity score is calculated by summing the severity scores for items 1–20. A total symptom score can also be calculated per cluster; re-experiencing (items 1–5), avoidance (items 6 and 7), negative alterations in cognitions and mood (items 8–17), and hyperarousal (items 15–20). A symptom cluster score may also be calculated for dissociation by summing items 29 and 30. To determine the PTSD diagnostic status, individual symptoms should be dichotomized as “present” or “absent”. A symptom is considered present only if the corresponding item severity score is rated 2 (= moderate/threshold or higher). Items 9 and 11–20 have the additional requirement of a trauma-relatedness rating of definite or probable. The DSM-5 diagnostic rule requires the presence of at least one criterion B symptom, one criterion C symptom, two criterion D symptoms, and two criterion E symptoms. Also, criteria F (disturbance at least 1 month) and G (disturbance that causes either clinically significant distress or functional impairment) must be met. Reliability of the CAPS-CA in a Dutch sample for the total scale ranged from coefficient *a* of 0.77 to .86, and the interrater reliability of the total scale is 0.99. Caps-CA also demonstrated divergent, convergent, and discriminant validity [57].

The Children’s Revised Impact of Event Scale [58] is translated in Dutch [59] and is a questionnaire for the assessment of traumatic impact across different types of trauma and life-threatening events. Children and parents will rate 13 items according to the frequency of their occurrence in the past 24 h in relation to the traumatic experiences (1 = none, 2 = rarely, 3 = sometimes, and 4 = often). Example items are: “Do you think about the ……. even when you don’t mean to?”, and “Do you avoid talking about …?” The sum score is used as an indicator of traumatic impact (ranging from 0 to 65). A score of 30 and over has been suggested as the most efficient cut-off for discriminating heightened risk for PTSD. Within a semi-clinical study population, Cronbach’s *a* was .89 for the 13-items child version [60].

The Trauma Symptom Checklist for Children [61], Dutch translation: *Trauma Symptoom Controle Lijst voor* Kinderen [62], is a questionnaire to assess the self-reported posttraumatic stress symptoms in children (8–12 years). It consists of 54 items clustering in eight scales: two validity scales (under response, hyper response) and six clinical scales (anxiety (9 items), depression (9 items), post-traumatic stress disorder (10 items), dissociation (10 items), anger (9 items), and sexual concerns (10 items)). A 4-point severity rating scale is used for all symptoms, ranging from 0 (= absent) to 3 (= very often). An example item is “feeling *afraid something bad might happen”.* Reliability was high for all subscales, with a Cronbach alpha ranging from 0.72 to 0.83 in a large sample [63].

##### Emotional security and physical safety

To assess the severity and intensity of the violence that children have been exposed to, a combined measure will be used including items from the Conflict Tactics Scale (78 items, 8-point rating scale) [64], the Conflict Tactics Scale parent child (27 and 39 items, 8-point rating scale) [64], the Parents Report of Traumatic Impact (21 items ) [65], and the Adverse Childhood Experience Questionnaire (10 items, 5-point rating scale) [66]. An example item is *I punched or hit my partner with something that could hurt*. This combined questionnaire has been used in a previous study on the effectiveness of a psycho-educational prevention program for children exposed to IPV [67]. The questionnaire covers the topics and duration of the violence and the nature of the arguments in the relationship with the (ex-partner), followed by items from the Conflict Tactics Scale parent child and Parents Report of Traumatic Impact about problems between parent and child, and traumatic events the child has experienced. The questionnaire also includes items about traumatic experiences in parents’ own childhood. A short questionnaire of 8 items for parent and child is added to assess if any new IPV incidents or other stressful events occurred.

To assess emotional security, the Dutch translation of the Security in the Interparental Subsystem [68] will be used. The Security in the Interparental Subsystem consists of 43-items with a 4-point ordinal scale (1 = not at all true of me, 4 = very true of me). Children are asked to answer questions about previous violence and conflicts between parents and current violence and conflicts between parents and partners. It has three scales and seven subscales: emotional reactivity (emotional reactivity (9 items), behavioural dysregulation (3 items), regulation of exposure to affect avoidance (7 items), involvement (6 items)) and internal representations (constructive family representations (4 items), destructive family representations (4 items), and conflict spillover representations (4 items)). An example item is *When my parents have an argument the family is still able to get along with each other*. The subscales show satisfactory internal consistency and test-retest reliability, and previous research supported the validity of the SIS scale [68]. Children will be asked to answer the same questions with respect to past fights and arguments between their parents and current fights and arguments between their mother and partner at T0, T12/14, and T13.

The Security in the Family System scale [69] will be used to assess how much children perceive their families as a reliable source of protection, stability, and support. The subscale “Secure” will be used, which assesses a secure pattern of emotional security. Children indicate the extent to which they agree with 7 statements using a 4-point scale ranging from “completely disagree” (1) to “completely agree” (4). An example item is *It’s worth caring about family members, even when things go wrong*. Psychometric properties of this security subscale are good, Cronbach’s *a* = 0.85 and test-retest reliability = 0.82 [69].

Caregivers report on children’s emotional security, including their emotional reactivity, behavioural involvement, and avoidance, after witnessing arguments between their parents using the Dutch translation of the Security in the Marital Subsystem Scale (SIMS) [68]. Items are completed on a 5-point ordinal scale from 1 (not at all like him/her) to 5 (a whole lot like him/her) and include feeling sad, angry, afraid, and upset (e.g. “Still seems upset after we argue”). The SIMS has been found reliable and has demonstrated discriminant, convergent, and predictive validity [70].

The Security Scale [71, 72] is a 15-item self-report questionnaire for children between the ages of 8 and 18 and measures self-reported attachment security with their parents [73]. Children and parents (independently) report on responsiveness and availability of the parent in the parent-child relationship (attachment). Each item is rated on a 5-point Likert scale (1 = strongly agree, 5 = strongly disagree). An example item is *I feel my mother really understands me*. Several studies indicate adequate reliability and validity [74]. High internal consistencies for 10 and 12-year-olds (*α* = 0.82, *α* = 0.79) are reported [75]. Test-retest reliability over a 2-week period was high (*r* = 0.75) [73]. The Security Scale is related to other attachment measures [75]. In a Dutch sample, internal consistency for the child version was *α* = 0.81 [76].

The Family Interaction Task (FIT) [77] is an observational instrument that measures parent-child interaction and consists of four tasks in which parent and child are instructed to complete a series of interactive tasks together. The FIT consists of four structured tasks: (1) guessing words by getting clues, (2) etch a sketch, (3) conflict task, and (4) completing a difficult but solvable puzzle by untangling a fixed ring from a standard within 4 min. These tasks are designed to elicit variations in parental and adolescent autonomy-promoting behaviours and emotional affect. The tasks are originally developed for children in middle childhood, but the tasks have been adapted for adolescents. Rating scales range from 1 to 5 and a higher score indicates a greater presence of that particular construct. Ratings are based on the complete session. Each videotaped session is independently coded by two coders (researchers). Interrater reliability was adequate (*n* = 101, ICC range .87–.94) in a Dutch sample [76].

#### Secondary outcomes

##### Child functioning

The Strengths and Difficulties Questionnaire (SDQ) [78] is a 25-item questionnaire using a 3-point scale (0 = not true, 2 = very true). The SDQ contains five subscales and one total scale. Subscales are (a) conduct problems (5 items), (b) emotional functioning (5 items), (c) hyperactivity/inattention (5 items), (d) peer problems (5 items), and (e) prosocial behaviour (5 items). There is a self-report and parent version. An example item is *I worry a lot*. The internal consistency of the teacher version is good. The parent and self-report versions have an internal consistency that is generally acceptable in Dutch samples [79]. In a Dutch sample, Cronbach’s alphas were between 0.76 and 0.79 for internalizing problems and between 0.82 and 0.85 for externalizing problems [80].

The Children’s Depression Inventory 2 (CDI2) [81] is a 28-item self-rated questionnaire that measures the symptoms of depression in children (7–18 years) with four subscales: negative mood/physical symptoms (9 items), negative self-esteem (6 items), interpersonal problems (5 items), and ineffectiveness (8 items). Per item, the child is asked to choose one of three sentences that best fits his/her feelings and thoughts in the past two weeks (e.g. *I am sad all the time*). The answers are calculated in a total score (ranging from 0 to 54). The internal consistency in a Dutch sample was high (*a* = 0.79), just as the test-retest reliability (*r* = 0.79) [79]. The CDI has high criterion validity and scores on the CDI correlate high with scores on other measures for depression [79].

The Child Dissociation Checklist (CDC) [82] is a 20-item parent-rated questionnaire with a 3-point Likert scale (0 = not true, 2 = very true). The child dissociation checklist is a screening instrument for dissociative problems in children (5–18 years). An example item is *Child does not remember or denies traumatic or painful experiences that are known to have occurred*. In a sample of maltreated and non-maltreated children, Cronbach’s alpha was 0.89 [83]. Discriminant validity is high [82].

##### Parent functioning

To assess parental stress, the Nijmegen Ouderlijke Stress Index—short version (NOSI-K) [84] will be used. The NOSI-K is a short version of the Parenting Stress Index [85] and a 25-item parent-rated questionnaire. The NOSI-K measures the stress related to the parent-child relationship and uses a 6-point scale (1 = totally disagree, 6 = totally agree). An example item is *I often have the feeling I cannot handle things very well*. Cronbach’s alpha for mothers and fathers is .90 and .91, respectively [86], and the NOS-K has moderate concurrent, discriminate, and criterion validity [84, 87].

To measure caregivers’ trauma knowledge, we will use a short self-report questionnaire developed for the training “Caring for Traumatized Children” [88]. The questionnaire comprises 6 items that reflect the six primary training goals, rated at a 10-point scale (1 = not accomplished, 10 = totally accomplished). Higher scores indicate higher trauma knowledge. An example item is *You know what the impact of trauma is on the development and behaviour of your child*.

Trauma symptoms in caregivers will be assessed with the Impact of Events Scale – Revised (IES-R) [89]. The Dutch version “Schokverwerkingslijst (SVL-22)” was developed by [90]. This questionnaire consists of 22 items measuring symptoms of PTSD during the last week. The SVL-22 measures three dimensions: intrusion (9 items), avoidance (8 items), and hyper-arousal (6 items). Parents rate the items on a 5-point Likert scale ranging from “not at all” (0) to “extremely” (4). An example item is *Any reminder brought back feelings about it*. Internal consistency was high (alpha = .88) [91]. Support construct validity is found in a Dutch study [90].

The Young Adult Self-Report [92] will be used to assess the psychopathology symptoms in parents. The short version of 29 items will be used in this study with items rated on a 3-point scale (0 = not true, 2 = often true). An example item is *I am too dependent on others*. The Dutch version has good reliability and validity [93]. In a Dutch study, Cronbach’s alpha was 0.92 [23].

The Daily Psychological Availability Scale [94] will be used to assess the parental availability for the child eight adapted items of the Daily Psychological Availability Scale. Items are measured using 7-point scales (1 = totally disagree, 7 = totally agree). An example item is *When I spent time with my oldest child after work today, I was not able to focus on my child*. A higher score on this scale represents more psychological availability for the child. Cronbach’s alpha was 0.78 for both fathers and mothers [94].

#### Participant timeline {13} (Fig. 1)

**Fig. 1:**
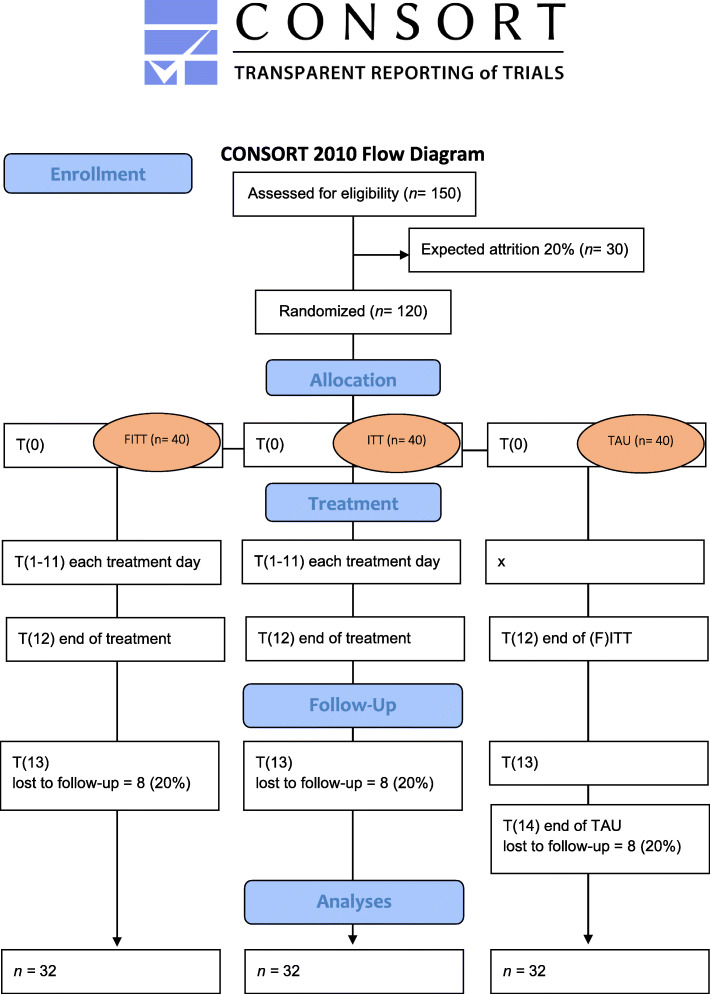
Consort diagram

#### Sample size {14}

Power analysis was conducted to determine whether an informative trial could be conducted given limitations imposed upon the maximum sample due to the duration of the study and size of the population of children indicated for trauma treatment, using G*power 3 [95]. Sample size calculation was made based on the assumptions of the primary objective and the first secondary objective. A priori, we decided that adding systemic intervention elements to individual trauma therapy would be justified if it would mean that on average at least one out of five children would have clinically significant benefit when given the full FITT compared to treatment without systemic elements (number needed to treat = 5; equivalent to *f* = 0.18).

For primary objective 1 (Does intensive trauma therapy with a family-based component (FITT) lead to a stronger reduction of trauma symptoms and stronger increase of structural emotional safety than intensive trauma therapy without this family-based component (ITT)?), the sample size was calculated based on between 2 groups by within effect with at least two measurements (pre, end/follow-up), setting *f* = 0.18 with a power of 80% and a significance level of 5% (two-sided). Default settings were used for the correlation among repeated measures (= 0.5) and non-sphericity correction (*ε* = 1). According to the power analysis, a total sample size of 64 children is required.

Secondary objective 1 (Does intensive trauma therapy with a family-based component (FITT) lead to faster reduction of trauma symptoms and increase of structural emotional safety than low-frequency trauma treatment with parent therapy and family sessions?) is based on two measurements (pre and intermediate/follow-up). In accordance with the first sample size calculation, we tested with setting *f* = 0.18 with a power of 80%, a significance level of 5% (two-sided) was used. Again, default settings were used for correlation among repeated measures (= 0.5) and non-sphericity correction (*ε* = 1). Based on this calculation, a total sample size of 64 children is required.

The FITT sample we use to test our first hypothesis will also be used to test the second hypothesis. Taking into account loss to follow-up and measurement unreliability (20%), we set the target sample size of the multicentre randomized controlled trial on *n* = 120. Based on the number of families treated in previous years, and based on the current intakes, the recruitment of 120 families that meet the inclusion criteria seems feasible.

#### Recruitment {15}

Recruitment will take place in four mental health centres for traumatized children in the Netherlands: Child and Youth Trauma Centre Kenter Jeugdhulp, Arq, UMC Utrecht, and GGZ Rivierduinen. Children and their parents are informed about the study by their therapists and will receive written information. After their permission, contact details of potential participants are provided to the researchers, who will contact the children and parents separately to provide more details about participation in the study. Children and their parents who are eligible for the trial are asked for informed consent. To reach the targeted sample size, brochures about the study are handed out to referrers and are available for families and therapists at sites.

## Assignment of interventions: allocation

### Sequence generation {16a}

Random allocation to either condition is needed, in order to reduce selection bias and to be able to attribute effects to parenting training and family sessions added upon an intensive trauma therapy. After informed consent is provided, families will be randomly assigned within each site to one of the treatment conditions. Within the multicentre trial, families are randomized by an independent researcher, following a computer-generated list with each participant having a 33.3% chance of being assigned to FITT, ITT, or TAU. The results of the randomization are communicated to the site per participating family.

### Concealment mechanism {16b}

Participating families are randomized by an independent researcher, following a computer-generated list with each participant having a 33.3% chance of being assigned to FITT, ITT, or TAU. The results of the randomization are provided to the centre per participating family. Subsequently, the measures and intervention will start.

### Implementation {16c}

An independent researcher will generate the allocation and directly informs the treatment team by e-mail about the treatment arm that families receive according to the randomization list. The therapist informs the families.

## Assignment of interventions: blinding

### Who will be blinded {17a}

Until randomization, both families and therapists are blind to the treatment allocation to mitigate bias due to expectation or disappointment. Nobody will be blinded during the intervention.

### Procedure for unblinding if needed {17b}

The design is open-label, and only data analysts will be blinded. Unblinding will therefore not occur.

### Data collection and management

#### Plans for assessment and collection of outcomes {18a}

The instruments with the best psychometric qualities and sensitivity to change in a short time period are selected for this study. For the primary outcomes different types of measurements are included: questionnaires for multiple informants, an interview and an observation task. A description of the study instruments can be found in the “Outcomes {12}” section. For this study, researchers will be trained and certified in the CAPS-CA interview and scoring protocol. The researchers will write a report based on the interview part of the CAPS-CA, and other answers are entered in a SPSS file. All data collected by questionnaires will be collected through an online survey system, Survalyzer, minimizing entry errors. Data will be stored and coded in a SPSS file. The FIT observation tasks will be administered according to a protocol and the recoded tasks will be coded by trained researchers with the use of a standard scoring form. For both the CAPS-CA and the FIT observation task, inter-rater reliability will be calculated for 20 participants. All data will be cleaned before analyses (e.g. checking on double entries, ranges, checking dates of data collections with treatment dates, coding missing data) in IBM SPSS.

#### Plans to promote participant retention and complete follow-up {18b}

It is common in trauma treatment that clients show avoidance behaviour because of the confrontation with anxiety-provoking details of the trauma. To prevent drop out psycho-education about avoidance behaviour and the disadvantages of avoiding is offered during treatment. To detect drop out of selective groups, participants who discontinue the treatment program will be asked to continue measurements.

#### Data management {19}

All research data will be handled confidentially and will be stored in closed facilities, which can only be assessed by researchers. The research data is confidential and will not be provided to third parties. Privacy of the participants will be protected by allocating key codes to the personalia, which will only be traceable with a separate list. The key codes are 3-digit numbers ranging from 000 to 150 and will be given in consecutive order. This list with personalia (names, addresses, phone numbers) that connects the participants with the research data is exclusively accessible by two of the researchers via an encrypted file and will eventually be destroyed. DarkStor from DANS (Dutch research data repository) will be used to store sensitive data and ArchStor (local data archive of Vrije Universiteit Amsterdam) for datasets that can be used for possible future research projects.

During data collection records of all specifics about the collections (e.g. measurements, dates, details about participants) will be kept in a logbook.

#### Confidentiality {27}

The research data will be stored and managed by the research team. All employees who work with confidential data will sign a confidentiality agreement, on which they state not to share the information with third parties. The digitalized research materials and confidentiality agreements will be stored in a secured folder on the network of VU Amsterdam. Data will be stored for 15 years, according to Medical Ethical Committee guidelines or for 5 years after the last publication based on this data.

#### Plans for collection, laboratory evaluation, and storage of biological specimens for genetic or molecular analysis in this trial/future use {33}

No biological specimens will be collected or stored (see the “Additional consent provisions for collection and use of participant data and biological specimens {26b}” section).

## Statistical methods

### Statistical methods for primary and secondary outcomes {20a}

The measurement level of each variable is at least ordinal. We control for the duration and severity of family violence [96], child functioning, parental psychopathology [97], and new incidents of family violence as a priori potential confounders. Analyses will be performed using the latest version of the software package Statistical Package for the Social Sciences (SPSS). First, to determine whether changes on continuous measures are different per treatment, we use linear mixed models. Continuous measures with only one time point will be analysed with independent samples *T*-test. Changes in categorical outcomes over the course of treatment across the conditions are tested with generalized estimating equations (GEE). To handle missing data, we will use maximum likelihood estimation within the linear mixed model analyses. For the planned GEE, we will explore the possibilities to deal with missing data, depending on the amount, randomness, and type of data missing. Analyses will primarily be performed according to the principles of intention-to-treat; however, they will be repeated as per-protocol analyses (see Table 4).

**Table 4:** Statistical analyses

*Statistical analyses*					
	Measure	Type of data	Timepoints	Statistical analysis	Subjects
Child PTSD	CAPS-CA	Categorical	3	GEE	ITT/PP
	CRIES	Continuous	4	Mixed models	ITT/PP
	TSCC	Continuous	3	Mixed models	ITT/PP
Child functioning	SDQ	Continuous	3	Mixed models	ITT/PP
	CDI	Continuous	3	Mixed models	ITT/PP
	CDC	Continuous	3	Mixed models	ITT/PP
Parent functioning	NOSI-K	Continuous	4	Mixed models	ITT/PP
	Trauma-informed	Continuous	3	Mixed models	ITT/PP
	TSI	Continuous	3	Mixed models	ITT/PP
	YAS-R	Continuous	3	Mixed models	ITT/PP
	DPAS	Continuous	3	Mixed models	ITT/PP
Structural emotional and physical safety	CTS	Continuous	3	Mixed models	ITT/PP
	CTS-pc	Continuous	3	Mixed models	ITT/PP
	PRTI	Continuous	3	Mixed models	ITT/PP
	ACE-Q	Continuous	3	Mixed models	ITT/PP
	New IPV incidents	Continuous	4	Mixed models	ITT/PP
	SIS	Continuous	3	Mixed models	ITT/PP
	SIFS	Continuous	3	Mixed models	ITT/PP
	SIM-PR	Continuous	3	Mixed models	ITT/PP
	Security scales	Continuous	3	Mixed models	ITT/PP
	FIT	Categorical	1	Independent samples T-test	ITT/PP

Descriptive and exploratory analyses will be carried out by standard methods. All continuous scores will be examined for normality. If variable distributions deviate too much from normality, corrective steps will be taken either by transforming variables or by using non-parametric tests. We will control for child functioning, parental psychopathology, duration, severity, and intensity of interparental violence, and new IPV incidents. The variables are treated as covariables in the analyses mentioned below.

To examine our primary hypothesis, linear mixed models will be used in case of continuous data. GEE will be used for the categorical data. We will use the end of treatment (T12/T14) and intermediate/follow-up time-point (T13) to compare FITT with ITT (objective 1).

To examine our secondary hypotheses, linear mixed models will be used. We will use the end of treatment time-point (T12/T14) to compare FITT with TAU. Control measures are child functioning; parental psychopathology; duration, severity, and intensity of interparental violence; and new IPV incidents. The variables are treated as covariables in the up-mentioned analyses. We will use the Benjamini-Hochberg method to correct for multiple comparisons.

### Interim analyses {21b}

No interim analysis will be performed because there are no anticipated problems that are detrimental to the participants.

### Methods for additional analyses (e.g. subgroup analyses) {20b}

There is no intention to perform additional analyses.

### Methods in analysis to handle protocol non-adherence and any statistical methods to handle missing data {20c}

If children and families drop out from treatment, we will try to continue measurements according to the randomized treatment condition and analyses will primarily be performed according to the principles of intention-to-treat. This means families are analyzed as participants of the treatment condition they are randomized to. However, analyses will be repeated as per-protocol analyses, which means that families are analysed as participants of the condition they actually received.

### Plans to give access to the full protocol, participant level-data and statistical code {31c}

With this publication, public access to the full protocol is granted. Because of privacy regulation and data protection, the participant-level dataset is not accessible. Statistical code will be available upon request.

### Oversight and monitoring

#### Composition of the coordinating centre and trial steering committee {5d}

Daily monitoring of the study will be done by the coordinating investigator, project leader, and principal investigator. Frequent contacts with the four trauma centres and research team meetings will be held to evaluate the progress of the study. All participating trauma centres have a contact person, who will be involved in all phases of the research. Furthermore, an independent physician of each participating centre monitors the trial on-site and randomly check the research of participants. Participants can consult an independent expert with questions about the trial.

#### Composition of the data monitoring committee, its role and reporting structure {21a}

To monitor the collection, storage, and processing of the data, peer auditors trained in data management will randomly check the research file on the following:

- Presence of informed consents
- Inclusion pace and drop out percentage
- Presence and completeness of the research data

If any errors are observed, the files of all study participants within the specific centre will be checked.

The presence and completeness of the research data will also be done on a daily basis by the research team.

#### Adverse event reporting and harms {22}

Adverse events are defined as any undesirable experience occurring to a subject during the study, whether or not considered related to the intervention. All adverse events reported spontaneously by the subject or observed by the investigator or the research team will be recorded. Should a child or a parent seem adversely affected by the questionnaires, interviews or observational task as observed by the researcher or therapist or as reported by the parents or children themselves, it may be decided to (temporarily) discontinue participation in the project. The investigator will report all SAEs to the sponsor without undue delay after obtaining knowledge of the events.

The sponsor will report the SAEs through the web portal *ToetsingOnline* to the accredited Medical Ethical Committee that approved the protocol, within 7 days of first knowledge for SAEs that result in death or are life-threatening followed by a period of a maximum of 8 days to complete the initial preliminary report. All other SAEs will be reported within a period of maximum 15 days after the sponsor has first knowledge of the serious adverse events.

All AEs will be followed until they have abated, or until a stable situation has been reached. Depending on the event, follow-up may require additional tests or medical procedures as indicated, and/or referral to the general physician or a medical specialist.

#### Frequency and plans for auditing trial conduct {23}

The investigators as well as peer auditors trained in data management will randomly check the research file. Both are not independent of the sponsor. The investigators will check the research file on a daily basis and the peer auditors will monitor the file once a year. Twice a year, the trial will also be monitored by the independent physician of each of the four participating youth mental health centres.

#### Plans for communicating important protocol amendments to relevant parties (e.g. trial participants, ethical committees) {25}

Since a pilot with the treatment protocol has been done before data collection starts, we do not expect important protocol modifications during the period of data collection. If any substantial modifications are necessary they will be first proposed to the Medical Ethical Committee and only after approval is implemented. If according to the therapist immediate care is necessary in cases of concerns about the safety of the child and/or families modifications can be implemented directly.

#### Dissemination plans {31a}

After data collection, the first results will be presented to experienced experts with a history of family violence to interpret the results of the trial. In a second expert meeting participants from the trial, professionals with a history of family violence and professionals in the field of family violence will be invited to discuss: (1) if and how FITT should be adjusted to special target groups like refugees, veterans, mentally retarded, different cultural backgrounds; (2) how FITT can be disseminated in the Netherlands and what is necessary for the implementation to achieve a nationwide network; and (3) how FITT can be processed in clinical guidelines in consultation with professional associations like the Dutch Society on Traumatic Stress.

A handbook for therapists about FITT will be published so the knowledge can be transferred, implemented, and executed in a sustainable way nationwide. The guide will provide information about the organization and structure of the program, and the questionnaires necessary to monitor FITT. We will develop a training for practitioners. Next, if proven effective, FITT is submitted to the Netherlands Youth Institute to assess the intervention for the database of effective interventions. Scientific articles about the research results will be submitted to national and international journals.

## Discussion

Family violence is a common problem [1, 2] that not only has a strong adverse impact on children [3–5], but it also has an indirect effect through the association of family violence with parenting and the parent-child relationship [5–8, 23]. The influence of family violence on children, parents, and thereby on parenting and the parent-child relationship makes family violence a complex problem difficult to recover from [9, 10]. Family-based intensive trauma treatment (FITT) addresses the factors on an individual and family level in a short period of time. This randomized controlled multicentre study tests if FITT is an effective treatment for concurrent reduction of trauma symptoms, improvement of parenting functioning, and increasing emotional and physical security in children, through the improvement of parent-child relationships—in order to establish long-term effects on the reduction of trauma symptoms and continuation of security in the family. In this study, FITT will be compared with an intensive trauma treatment without a parent and systemic component (ITT) and with treatment as usual (TAU, low-frequency trauma treatment with parent therapy and family sessions). Comparing these interventions with and without specific components can help to understand if and how these interventions work and what is necessary to increase treatment effects.

In this study, different therapists across multiple treatment centres are involved. To increase the internal reliability of the study, all therapists receive training and supervision during data collection. Because of the intensive character of the two treatment conditions, it could be not all eligible families want to participate in the study. To understand the effects under different conditions and prevent selection bias, all participants are analyzed as participants of the treatment condition they are randomized to and they will be repeated as per-protocol analyses. To mitigate bias due to expectation or disappointment both families and therapists are blind to treatment allocation until randomization. Another risk is that focusing on anxiety-provoking details of the traumatic events could be experienced as too burdensome for the participants resulting in avoidance behaviour and higher dropout rates. Previous research however showed that in intensive trauma-focused treatment programs the dropout rate is low [32, 33] or comparable to other treatment conditions [30] and trauma-focused treatments are most effective [29].

This study will provide more insight into the added value of the parental and systemic component to an intensive trauma treatment and the added value of intensifying treatment. Furthermore, it can contribute to the ongoing discussion in the field of trauma treatment about the necessity of preceding the trauma-focused treatment with a stabilization phase [98]. The main goal of this study is that it can contribute to the ambition to stop, reduce, and limit the impact of family violence and create emotional and psychical safety for children in a sustainable way.

## Trial status

Trial NL8592, version 1.0.

Recruitment of families for this study started on March 15, 2021, and runs until June 15, 2023. We expect recruitment to be completed in the first trimester of 2023.

## Abbreviations

EST: Emotional security theory
PTS: Posttraumatic stress
FITT: Family-based intensive trauma treatment
ITT: Intensive trauma treatment
RCT: Randomized-controlled trial
GEE: Generalized estimating equations
LMM: Linear mixed models
EMDR: Eye movement desentisation reprocessing
TAU: Treatment as usual
TF-CBT: Trauma-focused cognitive behavioural therapy
SAEs: Serious adverse events
