# Correction to: effectiveness of a high-intensive trauma-focused, family-based therapy for youth exposed to family violence: study protocol for a randomized controlled trial

**DOI:** 10.1186/s13063-022-06074-6

**Published:** 2022-02-08

**Authors:** Valerie Fictorie, Caroline Jonkman, Margreet Visser, Marjolein Vandenbosch, Majone Steketee, Carlo Schuengel

**Affiliations:** 1 Children’s Trauma Centre of Kenter Youthcare, Amsterdam, The Netherlands; 2 Vrije Universiteit Amsterdam, Amsterdam, The Netherlands; 3 Verwey-Jonker Institute, Utrecht, The Netherlands

**Correction to: Jonkman 23:46 (2022)**

**https://doi.org/10.1186/s13063-021-05981-4**

Following the publication of the original article [1], we were notified that Valerie Fictorie was mistakenly mentioned as last author instead of first author.

The original article has been corrected.
